# The *Caenorhabditis elegans* Female-Like State: Decoupling the Transcriptomic Effects of Aging and Sperm Status

**DOI:** 10.1534/g3.117.300080

**Published:** 2017-07-26

**Authors:** David Angeles-Albores, Daniel H. W. Leighton, Tiffany Tsou, Tiffany H. Khaw, Igor Antoshechkin, Paul W. Sternberg

**Affiliations:** * Department of Biology and Biological Engineering, and Howard Hughes Medical Institute, Caltech, Pasadena, California 91125; † Department of Human Genetics, Department of Biological Chemistry, and Howard Hughes Medical Institute, University of California, Los Angeles, California 90095; ‡ Department of Biology and Biological Engineering, Caltech, Pasadena, California 91125

**Keywords:** epistasis, genetic interactions, aging, life cycle, RNA-seq, germline sex determination

## Abstract

Understanding genome and gene function in a whole organism requires us to fully comprehend the life cycle and the physiology of the organism in question. *Caenorhabditis elegans* XX animals are hermaphrodites that exhaust their sperm after 3 d of egg-laying. Even though *C. elegans* can live for many days after cessation of egg-laying, the molecular physiology of this state has not been as intensely studied as other parts of the life cycle, despite documented changes in behavior and metabolism. To study the effects of sperm depletion and aging of *C. elegans* during the first 6 d of adulthood, we measured the transcriptomes of first-day adult hermaphrodites and sixth-day sperm-depleted adults, and, at the same time points, mutant *fog-2(lf)* worms that have a feminized germline phenotype. We found that we could separate the effects of biological aging from sperm depletion. For a large subset of genes, young adult *fog-2(lf)* animals had the same gene expression changes as sperm-depleted sixth-day wild-type hermaphrodites, and these genes did not change expression when *fog-2(lf)* females reached the sixth day of adulthood. Taken together, this indicates that changing sperm status causes a change in the internal state of the worm, which we call the female-like state. Our data provide a high-quality picture of the changes that happen in global gene expression throughout the period of early aging in the worm.

Transcriptome analysis by RNA-seq (Mortazavi *et al.* 2008) has allowed for in-depth analysis of gene expression changes between life stages and environmental conditions in many species (Gerstein *et al.* 2014; Blaxter *et al.* 2012). *Caenorhabditis elegans*, a genetic model nematode with extremely well-defined and largely invariant development (Sulston and Horvitz 1977; Sulston *et al.* 1983), has been subjected to extensive transcriptomic analysis across all stages of larval development (Hillier *et al.* 2009; Boeck *et al.* 2016; Murray *et al.* 2012) and many stages of embryonic development (Boeck *et al.* 2016). Although RNA-seq was used to develop transcriptional profiles of the mammalian aging process soon after its invention (Magalhães *et al.* 2010), few such studies have been conducted in *C. elegans* past the entrance into adulthood.

A distinct challenge to the study of aging transcriptomes in *C. elegans* is the hermaphroditic lifestyle of wild-type individuals of this species. Young adult hermaphrodites are capable of self-fertilization (Sulston and Brenner 1974; Corsi *et al.* 2015), and the resulting embryos will contribute RNA to whole-organism RNA extractions. Most previous attempts to study the *C. elegans* aging transcriptome have addressed the aging process only indirectly, or relied on the use of genetically or chemically sterilized animals to avoid this problem (Murphy *et al.* 2003; Halaschek-Wiener *et al.* 2005; Lund *et al.* 2002; McCormick *et al.* 2012; Eckley *et al.* 2013; Boeck *et al.* 2016; Rangaraju *et al.* 2015). In addition, most of these studies obtained transcriptomes using microarrays, which are less accurate than RNA-seq, especially for genes expressed at low levels (Wang *et al.* 2014).

Here, we investigate what we argue is a distinct state in the *C. elegans* life cycle. Although *C. elegans* hermaphrodites emerge into adulthood replete with sperm, after ∼3 d of egg-laying the animals become sperm-depleted and can only reproduce by mating. This marks a transition into what we define as the endogenous female-like state. This state is behaviorally distinguished by increased male-mating success (Garcia *et al.* 2007), which may be due to an increased attractiveness to males (Morsci *et al.* 2011). This increased attractiveness acts at least partially through production of volatile chemical cues (Leighton *et al.* 2014). These behavioral changes are also coincident with functional deterioration of the germline (Andux and Ellis 2008), muscle (Herndon *et al.* 2002), intestine (McGee *et al.* 2011), and nervous system (Liu *et al.* 2013), changes traditionally attributed to the aging process (Golden and Melov 2007).

To decouple the effects of aging and sperm loss, we devised a two-factor experiment. We examined wild-type XX animals at the beginning of adulthood (before worms contained embryos, referred to as first-day adults) and after sperm depletion (6 d after the last molt, which we term sixth-day adults). Second, we examined feminized XX animals that fail to produce sperm but are fully fertile if supplied with sperm by mating with males (see Figure 1). We used *fog-2* null mutants to obtain feminized animals. *fog-2* is involved in germ-cell sex determination in the hermaphrodite worm and is required for sperm production (Schedl and Kimble 1988; Clifford *et al.* 2000). *C. elegans* defective in sperm formation will emerge from the larval stage as female adults. As time moves forward, these spermless worms only exhibit changes related to biological aging. As a result, *fog-2(lf)* mutants should show fewer gene changes during the first 6 d of adulthood compared with their egg-laying counterparts that age and also transition from egg-laying into a sperm-depleted stage.

**Figure 1:**
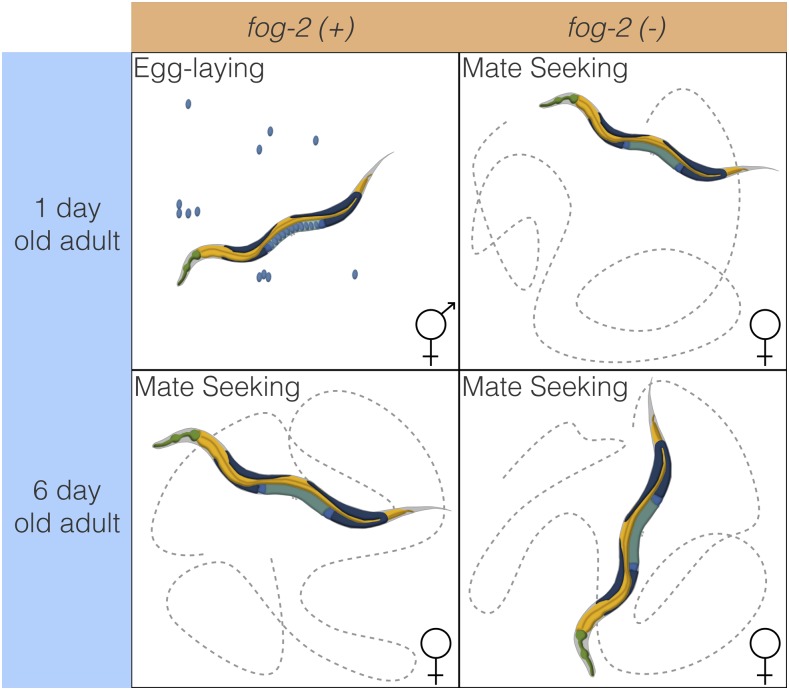
Experimental design to identify genes associated with sperm loss and aging. Studying the wild-type worm alone would measure time- and sperm-related changes at the same time, without allowing us to separate these changes. Studying the wild-type worm and a *fog-2(lf)* mutant would enable us to measure sperm-related changes but not time-related changes. By mixing both designs, we can measure and separate both modules.

Here, we show that we can detect a transcriptional signature associated with loss of hermaphroditic sperm marking entrance into the endogenous female-like state. We can also detect changes associated specifically with biological aging. Biological aging causes transcriptomic changes consisting of 5592 genes in *C. elegans*. 4552 of these changes occurred in both genotypes we studied, indicating they do not depend on sperm status. To facilitate exploration of the data, we have generated a website where we have deposited additional graphics, as well as all of the code used to generate these analyses: https://wormlabcaltech.github.io/Angeles_Leighton_2016.

## Materials and Methods

### Strains

Strains were grown at 20° on NGM plates containing *E. coli* OP50. We used the laboratory *C. elegans* strain N2 as our wild-type strain (Sulston and Brenner 1974). We also used the N2 mutant strain JK574, which contains the *fog-2(q71)* allele, for our experiments.

### RNA extraction

Synchronized worms were grown to either young adulthood or the sixth day of adulthood prior to RNA extraction. Synchronization and aging were carried out according to protocols described previously (Leighton *et al.* 2014). 1000–5000 worms from each replicate were rinsed into a microcentrifuge tube in S basal (5.85 g/liter NaCl, 1 g/liter ${\text{K}}_{2}{\text{HPO}}_{4},$ 6 g/liter ${\text{KH}}_{2}{\text{PO}}_{4}$), and then spun down at 14,000 rpm for 30 sec. The supernatant was removed and 1 ml of TRIzol was added. Worms were lysed by vortexing for 30 sec at room temperature and then 20 min at 4°. The TRIzol lysate was then spun down at 14,000 rpm for 10 min at 4° to allow removal of insoluble materials. Thereafter, the Ambion TRIzol protocol was followed to finish the RNA extraction (MAN0001271 rev. date: 13 Dec 2012). Three biological replicates were obtained for each genotype and each time point.

### RNA-seq

RNA integrity was assessed using an RNA 6000 Pico Kit for Bio-Analyzer (Agilent Technologies #5067–1513) and mRNA was isolated using a NEBNext Poly(A) mRNA Magnetic Isolation Module (New England Biolabs, NEB, #E7490). RNA-seq libraries were constructed using the NEBNext Ultra RNA Library Prep Kit for Illumina (NEB #E7530), following manufacturer’s instructions. Briefly, mRNA isolated from ∼1 μg of total RNA was fragmented to the average size of 200 nt by incubating at 94° for 15 min in first-strand buffer, cDNA was synthesized using random primers and ProtoScript II Reverse Transcriptase, followed by second-strand synthesis using Second Strand Synthesis Enzyme Mix (NEB). Resulting DNA fragments were end-repaired, dA-tailed and ligated to NEBNext hairpin adaptors (NEB #E7335). After ligation, adaptors were converted to the ‘Y’ shape by treating with USER enzyme, and DNA fragments were size-selected using Agencourt AMPure XP beads (Beckman Coulter #A63880) to generate fragment sizes between 250 and 350 bp. Adaptor-ligated DNA was PCR amplified, followed by AMPure XP bead clean-up. Libraries were quantified with Qubit dsDNA HS Kit (ThermoFisher Scientific #Q32854) and the size distribution was confirmed with High Sensitivity DNA Kit for Bioanalyzer (Agilent Technologies #5067–4626). Libraries were sequenced on Illumina HiSeq2500 in single-read mode with a read length of 50 nt, following manufacturer’s instructions. Base calls were performed with RTA 1.13.48.0 followed by conversion to FASTQ with bcl2fastq 1.8.4.

### Statistical analysis

#### RNA-seq analysis:

RNA-seq alignment was performed using Kallisto (Bray *et al.* 2016) with 200 bootstraps. Kallisto was run in single-end read mode, setting the average fragment length of 200bp, and a standard deviation of 60bp for all samples. Differential expression analysis was performed using Sleuth (Pimentel *et al.* 2016). The following general linear model (GLM) was fitted:

$$\log\left(y_{i}\right)=\beta_{0,i}+\beta_{G,i}\cdot G+\beta_{A,i}\cdot A+\beta_{A::G,i}\cdot A\cdot G,$$

where $y_{i}$ is the TPM count for the *i*th gene; $\beta_{0,i}$ is the intercept for the *i*th gene; $\beta_{X,i}$ is the regression coefficient for variable *X* for the *i*th gene; *A* is a binary age variable indicating first-day adult (0) or sixth-day adult (1); *G* is the genotype variable indicating wild type (0) or *fog-2(lf)* (1); and $\beta_{A::G,i}$ refers to the regression coefficient accounting for the interaction between the age and genotype variables in the *i*th gene. Genes were called significant if the FDR-adjusted q-value for any regression coefficient was <0.1. Our script for differential analysis is available on GitHub.

Regression coefficients and TPM counts were processed using Python 3.5 in a Jupyter Notebook (Pérez and Granger 2007). Data analysis was performed using the Pandas, NumPy and SciPy libraries (McKinney 2011; Van Der Walt *et al.* 2011; Oliphant 2007). Graphics were created using the Matplotlib and Seaborn libraries (Waskom *et al.* 2016; Hunter 2007). Interactive graphics were generated using Bokeh (Bokeh Development Team 2014).

Tissue, phenotype, and gene ontology enrichment analyses (TEA, PEA, and GEA, respectively) were performed using the WormBase Enrichment Suite for Python (Angeles-Albores *et al.* 2016, 2017a). Briefly, the WormBase Enrichment Suite accepts a list of genes and identifies the terms with which these genes are annotated. Terms are annotated by frequency of occurrence, and the probability that a term appears at this frequency under random sampling is calculated using a hypergeometric probability distribution. The hypergeometric probability distribution is extremely sensitive to deviations from the null distribution, which allows it to identify even small deviations from the null.

### Data availability

Strains are available from the *Caenorhabditis* Genetics Center. All of the data and scripts pertinent to this project, except the raw reads, can be found on our Github repository https://github.com/WormLabCaltech/Angeles_Leighton_2016. Supplementary Material, File S1 contains the list of genes that were altered in aging regardless of genotype. File S2 contains the list of genes and their associations with the *fog-2(lf)* phenotype. File S3 contains genes associated with the female-like state. Raw reads were deposited to the Sequence Read Archive under the accession code SUB2457229.

## Results and Discussion

### Decoupling time-dependent effects from sperm-status via GLMs

**Figure 2:**
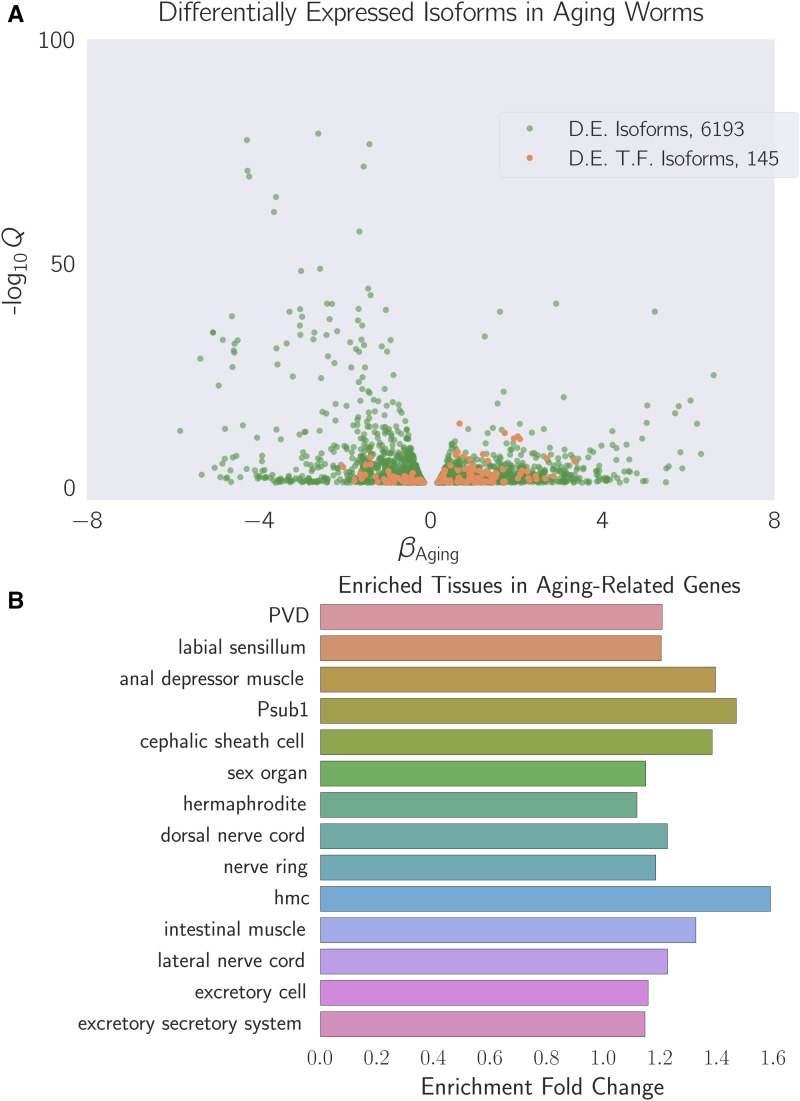
(A) Differentially expressed isoforms in the aging category. We identified a common aging expression signature between N2 and *fog-2(lf)* animals, consisting of 6193 differentially expressed isoforms totaling 5592 genes. The volcano plot is randomly down-sampled 30% for ease of viewing. Each point represents an individual isoform. $\beta_{\text{Aging}}$ is the regression coefficient. Larger magnitudes of *β* indicate a larger log-fold change. The *y*-axis shows the negative logarithm of the *q*-values for each point. Green points are differentially expressed isoforms; orange points are differentially expressed isoforms of predicted transcription factor genes (Reece-Hoyes *et al.* 2005). An interactive version of this graph can be found on our website. (B) Enriched tissues in aging-associated genes. Tissue enrichment analysis (Angeles-Albores *et al.* 2016) showed that genes associated with muscle tissues and the nervous system are enriched in aging-related genes. Only statistically significantly enriched tissues are shown. Enrichment fold change is defined as Observed/Expected. hmc, head mesodermal cell.

In order to decouple time-dependent effects from changes associated with loss of hermaphroditic sperm, we measured wild-type and *fog-2(lf)* adults at the first-day adult stage (before visible embryos were present) and sixth-day adult stage, when all wild-type hermaphrodites have laid all their eggs (see Figure 1), but mortality is still low (<10%) (Stroustrup *et al.* 2013). We obtained 16–19 million reads mappable to the *C. elegans* genome per biological replicate, which enabled us to identify 14,702 individual genes totaling 21,143 isoforms (see Figure 2A).

**Figure 3:**
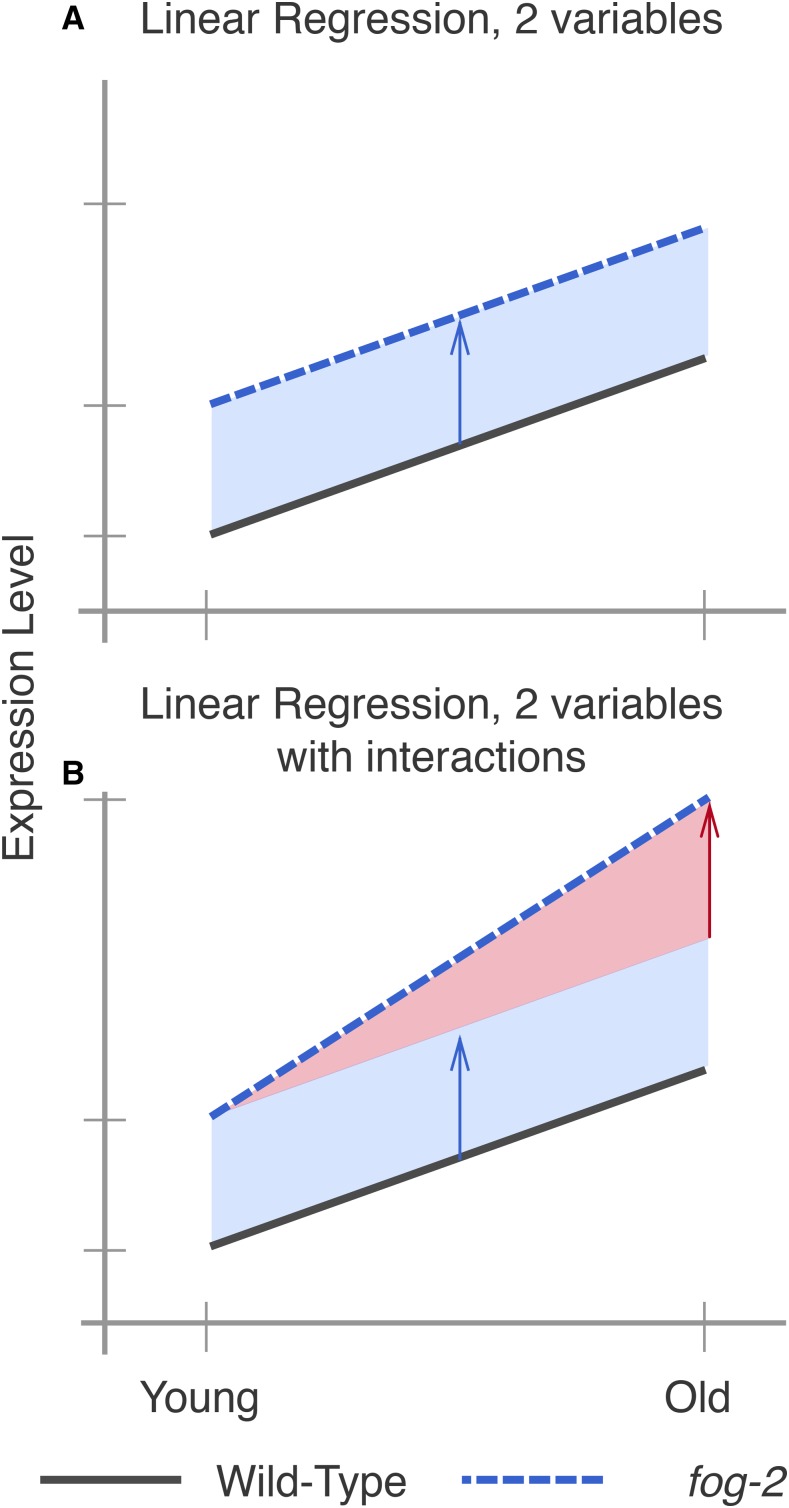
Explanation of linear regressions with and without interactions. (A) A linear regression with two variables, age and genotype. The expression level of a hypothetical gene increases by the same amount as worms age, regardless of genotype. However, *fog-2(lf)* has higher expression of this gene than the wild type at all stages (blue arrow). (B) A linear regression with two variables and an interaction term. In this example, the expression level of this hypothetical gene is different between wild-type worms and *fog-2(lf)* (blue arrow). Although the expression level of this gene increases with age, the slope is different between wild type and *fog-2(lf)*. The difference in the slope can be accounted for through an interaction coefficient (red arrow).

One way to analyze the data from this two-factor design is by pairwise comparison of the distinct states. However, such an analysis would not make full use of all the statistical power afforded by this experiment. Another method that makes full use of the information in our experiment is to perform a linear regression in three dimensions (two independent variables, age and genotype, and one output). A linear regression with one parameter (age, for example) would fit a line between expression data for young and old animals. When a second parameter is added to the linear regression, said parameter can be visualized as altering the y-intercept, but not the slope, of the first line (see Figure 3A). When the variables are categorical, as in this case, this model is called a General Linear Model (GLM).

Although a simple linear model is often useful, sometimes it is not appropriate to assume that the two variables under study are entirely independent. For example, in our case, three out of the four time point/genotype combinations we studied did not have sperm, and sperm status is associated with both the *fog-2(lf)* self-sterile phenotype and biological age of the wild-type animal. One way to statistically model such correlation between variables is to add an interaction term to the linear regression. This interaction term allows extra flexibility in describing how changes occur between conditions. For example, suppose a given theoretical gene *X* has expression levels that increase in a *fog-2*-dependent manner, but also increase in an age-dependent manner. However, aged *fog-2(lf)* animals do not have the expression levels of *X* that would be expected from adding the effect of the two perturbations; instead, the expression levels of *X* in this animal are considerably above what is expected. In this case, we could add a positive interaction coefficient to the model to explain the effect of genotype on the y-intercept as well as the slope (see Figure 3B). When the two perturbations affect a single genetic pathway, these interactions can be interpreted as epistatic interactions.

For these reasons, we used a GLM with interactions to identify a transcriptomic profile associated with the *fog-2(lf)* genotype independently of age, as well as a transcriptomic profile of *C. elegans* aging common to both genotypes. The change associated with each variable is referred to as *β*; this number, although related to the natural logarithm of the fold change, is not equal to it. However, it is true that larger magnitudes of *β* indicate greater change. Thus, for each gene we performed a linear regression, and we evaluated whether the *β* values associated with each coefficient were significantly different from 0 via a Wald test corrected for multiple hypothesis testing. A coefficient was considered to be significantly different from 0 if the q-value associated with it was <0.1.

### A quarter of all genes change expression between the 1st day of adulthood and the 6th day of adulthood in C. elegans

We identified a transcriptomic signature consisting of 5592 genes that were differentially expressed in sixth-day adult animals of either genotype relative to first-day adult animals (see File S2). This constitutes slightly less than a quarter of the genes in *C. elegans*. TEA (Angeles-Albores *et al.* 2016) showed that nervous tissues, including the “nerve ring”, “dorsal nerve cord”, “PVD”, and “labial sensillum”, were enriched in genes that become differentially expressed through aging. Likewise, certain muscle groups (“anal depressor muscle” and “intestinal muscle”) were enriched (see Figure 2B). GEA (Angeles-Albores *et al.* 2017a) revealed that genes that were differentially expressed during the course of aging were enriched in terms involving respiration (“respiratory chain” and “oxoacid metabolic process”); translation (“cytosolic large ribosomal subunit”); and nucleotide metabolism (“purine nucleotide”, “nucleoside phosphate”, and “ribose phosphate”). PEA (Angeles-Albores *et al.* 2017a) showed that this gene list was associated with phenotypes that affect the *C. elegans* gonad, including “gonad vesiculated”, “gonad small”, “oocytes lack nucleus”, and “rachis narrow”.

To verify the quality of our dataset, we generated a list of 1056 gold-standard genes expected to be altered in sixth-day adult worms using previous literature reports, including downstream genes of *daf-12*, *daf-16*, and aging and lifespan extension datasets (Murphy *et al.* 2003; Halaschek-Wiener *et al.* 2005; Lund *et al.* 2002; McCormick *et al.* 2012; Eckley *et al.* 2013). Of 1056 standard genes, we found 506 genes in our time-responsive dataset. This result was statistically significant, with a p-value <10^–38^.

Next, we used a published compendium (Reece-Hoyes *et al.* 2005) to search for known or predicted transcription factors. We found 145 transcription factors in the set of genes with differential expression in aging nematodes. We subjected this list of transcription factors to TEA to understand their expression patterns. Six of these transcription factors were expressed in the “hermaphrodite specific neuron”, a neuron physiologically relevant for egg-laying (*hlh-14*, *sem-4*, *ceh-20*, *egl-46*, *ceh-13*, *hlh-3*), which represented a statistically significant twofold enrichment of this tissue (*q* <10^–1^). The term “head muscle” was also overrepresented at twice the expected level (*q* <10^–1^, 13 genes).

### The whole-organism fog-2(lf) differential expression signature

We identified 1881 genes associated with the *fog-2(lf)* genotype, including 60 transcription factors (see File S3). TEA showed that the terms “AB”, “somatic gonad”, “uterine muscle”, “cephalic sheath cell”, “spermathecal-uterine junction”, and “PVD” were enriched in this gene set. The “somatic gonad” and “spermathecal-uterine junction” are both near the site of action of *fog-2(lf)* (the germline) and possibly reflect physiological changes resulting from a lack of sperm. PEA showed that only a single phenotype term, “spindle orientation variant” was enriched in the *fog-2(lf)* transcriptional signature (*q* <10^–1^, 38 genes, twofold enrichment). Most genes annotated as “spindle orientation variant” were slightly upregulated, and therefore are unlikely to uniquely reflect reduced germline proliferation. GO term enrichment was very similar to that of the aging gene set and reflected enrichment in annotations pertaining to translation and respiration. Unlike the aging gene set, the *fog-2(lf)* signature was significantly enriched in “myofibril” and “G-protein coupled receptor binding” (*q* <10^–1^). Enrichment of the term “G-protein coupled receptor binding” was due to 14 genes: *cam-1*, *mom-2*, *dsh-1*, *spp-10*, *flp-6*, *flp-7*, *flp-9*, *flp-13*, *flp-14*, *flp-18*, *K02A11.4*, *nlp-12*, *nlp-13*, and *nlp-40*. *dsh-1*, *mom-2* and *cam-1* are members of the Wnt signaling pathway. Most of these genes’ expression levels were upregulated, suggesting increased G-protein binding activity in *fog-2(lf)* mutants.

### The fog-2(lf) expression signature overlaps significantly with the aging signature

Of the 1881 genes that we identified in the *fog-2(lf)* signature, 1040 genes were also identified in our aging set. Moreover, of these 1040 genes, 905 genes changed in the same direction in response to either aging or germline feminization. The overlap between these signatures suggests an interplay between sperm status and age. The nature of the interplay should be captured by the interaction coefficients in our model. There are four possibilities. First, the *fog-2(lf)* worms may have a fast-aging phenotype, in which case the interaction coefficients should match the sign of the aging coefficient. Second, the *fog-2(lf)* worms may have a slow-aging phenotype, in which case the interaction coefficients should have an interaction coefficient that is of opposite sign, but not greater in magnitude than the aging coefficient (if a gene increases in aging in a wild-type worm, it should still increase in a *fog-2(lf)* worm, albeit to a lesser extent). Third, the *fog-2(lf)* worms may exhibit a rejuvenation phenotype. If this is the case, then these genes should have an interaction coefficient that is of opposite sign and greater magnitude than their aging coefficient, such that the change of these genes in *fog-2(lf)* mutant worms is reversed relative to the wild type. Finally, if these genes are indicative of a female-like state, then these genes should not change with age in *fog-2(lf)* animals, since these animals do not exit this state during the course of the experiment. Moreover, because wild-type worms become female as they age, a further requirement for a transcriptomic signature of the female-like state is that aging coefficients for genes in this signature should have genotype coefficients of equal sign and magnitude. In other words, entrance into the female-like state should be not be path-dependent.

**Figure 4:**
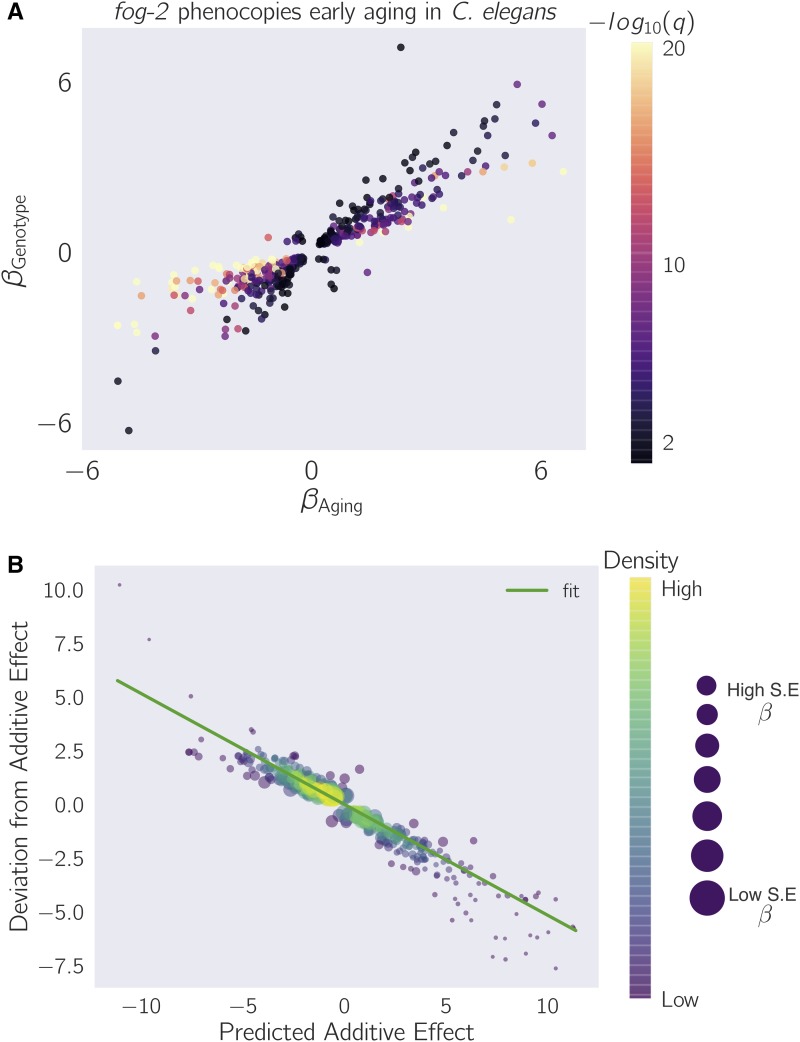
*fog-2(lf)* partially phenocopies early aging in *C. elegans*. The *β* on each axis is the regression coefficient from the GLM, and can be loosely interpreted as an estimator of the log-fold change. Loss of *fog-2* is associated with a transcriptomic phenotype involving 1881 genes. 1040/1881 of these genes are also altered in wild-type worms as they progress from young adulthood to old adulthood, and 905 change in the same direction. However, progression from young to old adulthood in a *fog-2(lf)* background results in no change in the expression level of these genes. (A) We identified genes that change similarly during feminization and aging. The correlation between feminization and aging is almost 1:1. (B) Epistasis plot of aging *vs.* feminization. Epistasis plots indicate whether two genes (or perturbations) act on the same pathway. When two effects act on the same pathway, this is reflected by a slope of –0.5. The measured slope was –0.51 ± 0.01.

**Figure 5:**
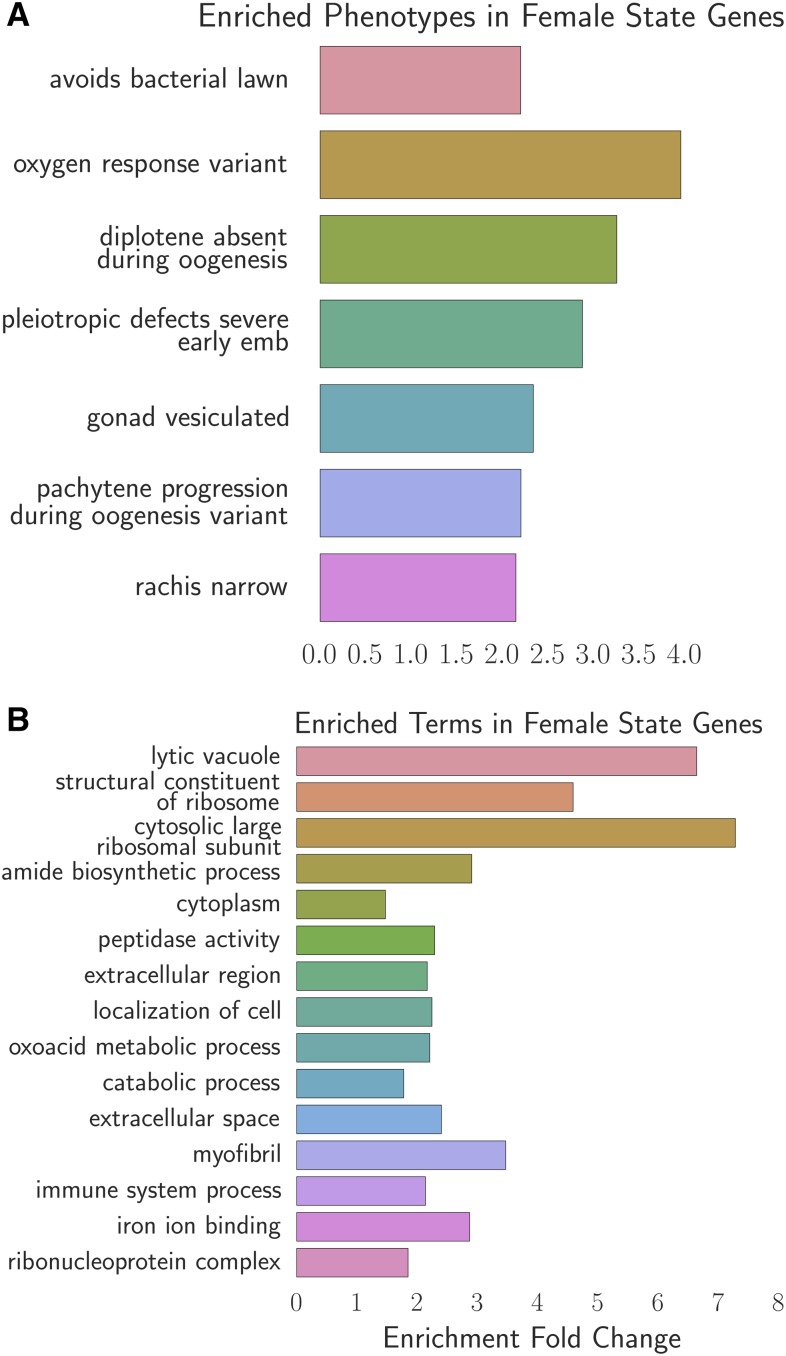
Phenotype and GO enrichment of genes involved in the female-like state. (A) Phenotype enrichment analysis. (B) Gene ontology enrichment analysis. Most of the terms enriched in PEA reflect the abundance of ribosomal subunits present in this gene set.

To evaluate which of these possibilities was most likely, we selected the 1040 genes that had aging, genotype, and interaction coefficients significantly different from zero, and we plotted their temporal coefficients against their genotype coefficients (see Figure 4A and Figure 5). We observed that the aging coefficients were strongly predictive of the genotype coefficients. Most of these genes fell near the line *y* = *x*, suggesting that these genes define a female-like state.

We considered how loss-of-function of *fog-2* and aging could both interact to cause entry into this state. We reasoned that a plausible mechanism is that *fog-2* promotes sperm production, and aging promotes sperm depletion. This simple pathway model suggests that a double perturbation consisting of aging and loss of function of *fog-2* should show nonadditivity of phenotypes (generalized epistasis). To test whether these two perturbations deviate from additivity, we generated an epistasis plot using this gene set. We have previously used epistasis plots to measure transcriptome-wide epistasis between genes in a pathway (Angeles-Albores *et al.* 2017b). Briefly, an epistasis plot shows the expected expression of a double perturbation under an additive model (null model) on the *x*-axis, and the deviation from this null model in the *y*-axis. In other words, we calculated the *x*-coordinates for each point by adding $\beta_{\text{Genotype}}+\beta_{\text{Aging}},$ and the *y*-coordinates are equal to $\beta_{\text{Interaction}}$ for each isoform. We have previously shown that if two genes or perturbations act within a linear pathway, an epistasis plot will generate a line with slope equal to –0.5. When we generated an epistasis plot and found the line of best fit, we observed a slope of –0.51 ± 0.01, which suggests that the *fog-2* gene and time are acting to generate a single transcriptomic phenotype along a single pathway. Overall, we identified 405 genes that changed in the same direction through age or mutation of the *fog-2(lf)* gene, and that had an interaction coefficient of opposite sign to the aging or genotype coefficient (see File S4). Taken together, these observations suggest that these 405 genes define a female-like state in *C. elegans*.

### Analysis of the female-like state expression signature

To better understand the changes that happen after sperm loss, we performed TEA, PEA, and GEA on the set of 405 genes that we associated with the female-like state (see Fig. 5). TEA showed no tissue enrichment using this gene set. GEA showed that this gene list was enriched in constituents of the ribosomal subunits almost four times above background (*q* <10^–5^, 17 genes). The enrichment of ribosomal constituents in this gene set in turn drives the enriched phenotypes: “avoids bacterial lawn”, “diplotene absent during oogenesis”, “gonad vesiculated”, “pachytene progression during oogenesis variant”, and “rachis narrow.” The expression of most of these ribosomal subunits is downregulated in aged animals or in *fog-2(lf)* mutants.

## Discussion

### Defining an early aging phenotype

Our experimental design enables us to decouple the effects of egg-laying from aging. As a result, we identified a set of almost 4000 genes that are altered similarly between wild-type and *fog-2(lf)* mutants. Owing to the read depth of our transcriptomic data (20 million reads) and the number of samples measured (three biological replicates for four different life stages/genotypes), this dataset constitutes a high-quality description of the transcriptomic changes that occur in aging populations of *C. elegans*. Although our data only capture ∼50% of the expression changes reported in earlier aging transcriptome literature, this disagreement can be explained by a difference in methodology; earlier publications typically addressed the aging of fertile wild-type hermaphrodites only indirectly, or queried aging animals at a much later stage of their life cycle.

### GLMs enable epistasis measurements

We set out to study the self-fertilizing (hermaphroditic) to self-sterile (female-like) transition by comparing wild-type animals with *fog-2(lf)* mutants as they aged. Our computational approach enabled us to distinguish between two biological processes that are correlated within samples. Because of this intrasample correlation, identifying this state via pairwise comparisons would not have been straightforward. Although it is a favored method among biologists, such pairwise comparisons suffer from a number of drawbacks. First, pairwise comparisons are unable to draw on the full statistical power available to an experiment because they discard almost all information except the samples being compared. Second, pairwise comparisons require a researcher to define *a priori* which comparisons are informative. For experiments with many variables, the number of pairwise combinations is explosively large. Indeed, even for this two-factor experiment, there are six possible pairwise comparisons. On the other hand, by specifying a linear regression model, each gene can be summarized with three variables, each of which can be analyzed and understood without the need to resort to further pairwise combinations.

### The C. elegans female-like state

**Figure 6:**
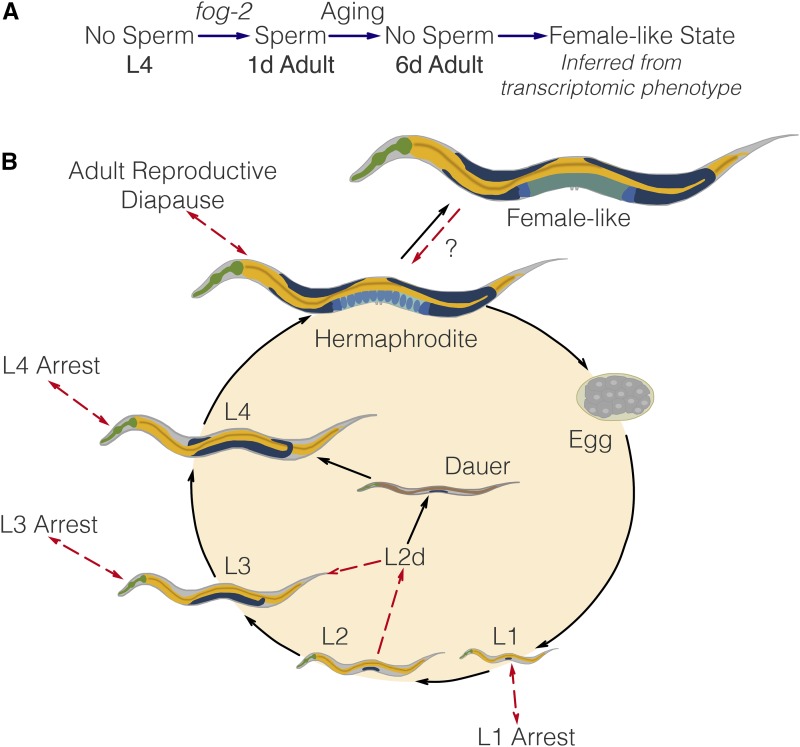
(A) A substrate-dependent model showing how *fog-2* promotes sperm generation, whereas aging promotes sperm depletion, leading to entry into the female-like state. Such a model can explain why *fog-2* and aging appear epistatic to each other. (B) The complete *C. elegans* life cycle. Recognized stages of *C. elegans* are marked by black arrows. States are marked by red arrows to emphasize that at the end of a state, the worm returns to the developmental time point it was at before entering the state. The L2d state is an exception. It is the only stage that does not return to the same developmental time point; rather, the L2d state is a permissive state that allows entry into either dauer or the L3 stage. We have presented evidence of a female-like state in *C. elegans*. At this point, it is unclear whether the difference between hermaphrodites and females is reversible by males. Therefore, it remains unclear whether it is a stage or a true state.

Our explorations have shown that the loss of *fog-2(lf)* partially phenocopies the transcriptional events that occur naturally as *C. elegans* ages from the first day of adulthood to the sixth day of adulthood. Moreover, epistasis analysis of these perturbations suggests that they act on the same pathway, namely sperm generation and depletion (see Figure 6). Self-sperm generation promotes the hermaphrodite state, whereas sperm depletion marks entry into the female-like state. Given the enrichment of neuronal transcription factors that are associated with sperm loss in our dataset, we believe this dataset should contain some of the transcriptomic modules that are involved in these pheromone production and behavioral pathways, although we have been unable to find these genes.

Behavioral and physiological changes upon mating are not unknown in other species. In particular, in the fruit fly *Drosophila melanogaster*, a sex peptide present in the male seminal fluid is known to drive changes in gene expression (Liu and Kubli 2003; Xue and Noll 2000; Avila *et al.* 2011; Heifetz *et al.* 2014; Rezával *et al.* 2014; Mack *et al.* 2006) as well as behavior. More recently, sperm was found to be necessary to drive changes in aggression in the fruit fly (Bath *et al.* 2017). These changes are often reversible upon the disappearance of seminal fluid or sperm. In the case of *C. elegans*, we have observed that sperm loss is associated with gene expression changes that probably reflect physiological changes in the worm. Our experimental design did not include a test for reversibility of these changes. The possibility of a rescue experiment with males raises interesting possibilities. What fraction of the changes observed upon loss of self-sperm are reversible? Does male seminal fluid or male sperm cause changes beyond rescue?

### The C. elegans life cycle, life stages and life states

*C. elegans* has a complicated life cycle, with two alternative developmental pathways that have multiple stages (larval development and dauer development), followed by reproductive adulthood. In addition to these developmental stages, researchers have recognized that *C. elegans* has numerous life states that it can enter into when given instructive environmental cues. One such state is the L1 arrest state, where development ceases entirely upon starvation (Johnson *et al.* 1984; Baugh and Sternberg 2006). More recently, researchers have described additional diapause states that the worm can access at the L3, L4, and young adult stages under conditions of low food (Angelo and Gilst 2009; Seidel and Kimble 2011; Schindler *et al.* 2014). Not all states of *C. elegans* are arrested, however (see Figure 6). For example, the L2d state is induced by crowded and nutrient-poor conditions (Golden and Riddle 1984). While in this state, the worm is capable of entry into either dauer or the L3 larval stage, depending on environmental conditions. Thus, the L2d state is a permissive state, and marks the point at which the nematode development is committed to a single developmental pathway.

Identification of the *C. elegans* life states has often been performed by morphological studies (as in the course of L4 arrest or L2d) or via time courses (L1 arrest). However, not all states may be visually identifiable, or, even if they are, the morphological changes may be very subtle, making positive identification difficult. However, the detailed information afforded by a transcriptome should in theory provide sufficient information to definitively identify a state, since transcriptomic information underlies morphology. Moreover, transcriptomics can provide an insight into the physiology of complex metazoan life states. By identifying differentially expressed genes and using ontology enrichment analyses to identify gene functions, sites of expression, or phenotypes that are enriched in a given gene set, we can obtain a clear picture of the changes that occur in the worm analogous to identifying gross morphological changes.

RNA-seq is a powerful technology that has been used successfully in the past as a qualitative tool for target acquisition, although recent work has also used RNA-seq to measure genetic interactions via epistasis (Dixit *et al.* 2016; Angeles-Albores *et al.* 2017b). Here, we have shown that whole-organism RNA-seq data can also be used to successfully identify internal states in a multicellular organism.

## Supplementary Material

Supplemental material is available online at www.g3journal.org/lookup/suppl/doi:10.1534/g3.117.300080/-/DC1.

## References

[bib1] Andux S. Ellis R. E., 2008 Apoptosis maintains oocyte quality in aging *Caenorhabditis elegans* females. PLoS Genet. 4: e1000295. 19057674 10.1371/journal.pgen.1000295 PMC2585808

[bib2] Angeles-Albores D. Lee R. Y. N. Chan J. Sternberg P. W., 2016 Tissue enrichment analysis for *C. elegans* genomics. BMC Bioinformatics 17: 366. 27618863 10.1186/s12859-016-1229-9 PMC5020436

[bib3] Angeles-Albores D. Lee R. Y. Chan J. Sternberg P. W., 2017 a Phenotype and gene ontology enrichment as guides for disease modeling in *C. elegans*. bioRxiv DOI: 10.1101/106369.

[bib4] Angeles-Albores D. Puckett Robinson C. Williams B. A. Wold B. J. Sternberg P. W., 2017 b Reconstructing a metazoan genetic pathway with transcriptome-wide epistasis measurements. bioRxiv DOI: 10.1101/112920. PMC5879656 29531064

[bib5] Angelo G. Gilst M. R. V., 2009 Cells and extends reproductive. Science 326: 954–958. 19713489 10.1126/science.1178343

[bib6] Avila F. W. Sirot L. K. LaFlamme B. A. Rubinstein C. D. Wolfner M. F., 2011 Insect seminal fluid proteins: identification and function. Annu. Rev. Entomol. 56: 21–40. 20868282 10.1146/annurev-ento-120709-144823 PMC3925971

[bib7] Bath E. Bowden S. Peters C. Reddy A. Tobias J. A., 2017 Sperm and sex peptide stimulate aggression in female *Drosophila*. Nat. Ecol. Evol. 1: 0154. 28580431 10.1038/s41559-017-0154 PMC5447820

[bib8] Baugh L. R. Sternberg P. W., 2006 DAF-16/FOXO regulates transcription of cki-1/Cip/Kip and repression of *lin-4* during *C. elegans* L1 arrest. Curr. Biol. 16: 780–785 16631585 10.1016/j.cub.2006.03.021

[bib9] Blaxter M. Kumar S. Kaur G. Koutsovoulos G. Elsworth B., 2012 Genomics and transcriptomics across the diversity of the Nematoda. Parasite Immunol. 34: 108–120. 22044053 10.1111/j.1365-3024.2011.01342.x

[bib10] Boeck M. E. Huynh C. Gevirtzman L. Thompson O. A. Wang G., 2016 The time-resolved transcriptome of *C. elegans*. Genome Res. 26: 1441–1450. 27531719 10.1101/gr.202663.115 PMC5052054

[bib11] Bokeh Development Team, 2014 Bokeh: Python library for interactive visualization. Available at: http://www.bokeh.pydata.org.

[bib12] Bray N. L. Pimentel H. J. Melsted P. Pachter L., 2016 Near-optimal probabilistic RNA-seq quantification. Nat. Biotechnol. 34: 525–527. 27043002 10.1038/nbt.3519

[bib13] Clifford R. Lee M. H. Nayak S. Ohmachi M. Giorgini F., 2000 FOG-2, a novel F-box containing protein, associates with the GLD-1 RNA binding protein and directs male sex determination in the *C. elegans* hermaphrodite germline. Development 127: 5265–5276. 11076749 10.1242/dev.127.24.5265

[bib14] Corsi A. K. Wightman B. Chalfie M., 2015 A transparent window into biology: a primer on *Caenorhabditis elegans*. Genetics 200: 387–407. 26088431 10.1534/genetics.115.176099 PMC4492366

[bib15] Dixit A. Parnas O. Li B. Chen J. Fulco C. P., 2016 Perturb-Seq: dissecting molecular circuits with scalable single-cell RNA profiling of pooled genetic screens. Cell 167: 1853–1866.e17. 27984732 10.1016/j.cell.2016.11.038 PMC5181115

[bib16] Eckley D. M. Rahimi S. Mantilla S. Orlov N. V. Coletta C. E., 2013 Molecular characterization of the transition to mid-life in *Caenorhabditis elegans*. Age 35: 689–703. 22610697 10.1007/s11357-012-9401-2 PMC3636400

[bib17] Garcia L. R. LeBoeuf B. Koo P., 2007 Diversity in mating behavior of hermaphroditic and male-female *Caenorhabditis* nematodes. Genetics 175: 1761–1771. 17277358 10.1534/genetics.106.068304 PMC1855125

[bib18] Gerstein M. B. Rozowsky J. Yan K.-K. Wang D. Cheng C., 2014 Comparative analysis of the transcriptome across distant species. Nature 512: 445–448. 25164755 10.1038/nature13424 PMC4155737

[bib19] Golden J. W. Riddle D. L., 1984 The *Caenorhabditis elegans* dauer larva: developmental effects of pheromone, food, and temperature. Dev. Biol. 102: 368–378. 6706004 10.1016/0012-1606(84)90201-x

[bib20] Golden, T. R., and S. Melov, 2007 Gene expression changes associated with aging in C. elegans. WormBook: the online review of *C. elegans* biology pp. 1–12. 10.1895/wormbook.1.127.2 PMC4781587 18050504

[bib21] Halaschek-Wiener J. Khattra J. S. McKay S. Pouzyrev A. Stott J. M., 2005 Analysis of long-lived *C. elegans* daf-2 mutants using serial analysis of gene expression. Genome Res. 15: 603–615. 15837805 10.1101/gr.3274805 PMC1088289

[bib22] Heifetz Y. Lindner M. Garini Y. Wolfner M., 2014 Mating regulates neuromodulator ensembles at nerve termini innervating the *Drosophila* reproductive tract. Curr. Biol. 24: 731–737. 24631240 10.1016/j.cub.2014.02.042 PMC4020355

[bib23] Herndon L. A. Schmeissner P. J. Dudaronek J. M. Brown P. a. Listner K. M., 2002 Stochastic and genetic factors influence tissue-specific decline in ageing *C. elegans*. Nature 419: 808–814. 12397350 10.1038/nature01135

[bib24] Hillier L. W. Reinke V. Green P. Hirst M. Marra M. A., 2009 Massively parallel sequencing of the polyadenylated transcriptome of *C. elegans*. Genome Res. 19: 657–666. 19181841 10.1101/gr.088112.108 PMC2665784

[bib25] Hunter J. D., 2007 Matplotlib: a 2D graphics environment. Comput. Sci. Eng. 9: 99–104.

[bib26] Johnson T. E. Mitchell D. H. Kline S. Kemal R. Foy J., 1984 Arresting development arrests aging in the nematode *Caenorhabditis elegans*. Mech. Ageing Dev. 28: 23–40. 6542614 10.1016/0047-6374(84)90150-7

[bib27] Leighton D. H. W. Choe A. Wu S. Y. Sternberg P. W., 2014 Communication between oocytes and somatic cells regulates volatile pheromone production in *Caenorhabditis elegans*. Proc. Natl. Acad. Sci. USA 111: 17905–17910. 25453110 10.1073/pnas.1420439111 PMC4273420

[bib28] Liu H. Kubli E., 2003 Sex-peptide is the molecular basis of the sperm effect in *Drosophila* melanogaster. Proc. Natl. Acad. Sci. USA 100: 9929–9933. 12897240 10.1073/pnas.1631700100 PMC187889

[bib29] Liu J. Zhang B. Lei H. Feng Z. Liu J., 2013 Functional aging in the nervous system contributes to age-dependent motor activity decline in *C. elegans*. Cell Metab. 18: 392–402. 24011074 10.1016/j.cmet.2013.08.007 PMC3811915

[bib30] Lund J. Tedesco P. Duke K. Wang J. Kim S. K., 2002 Transcriptional profile of aging in *C. elegans*. Curr. Biol. 12: 1566–1573. 12372248 10.1016/s0960-9822(02)01146-6

[bib31] Mack P. D. Kapelnikov A. Heifetz Y. Bender M., 2006 Mating-responsive genes in reproductive tissues of female *Drosophila melanogaster*. Proc. Natl. Acad. Sci. USA 103: 10358–10363. 16798875 10.1073/pnas.0604046103 PMC1502462

[bib32] Magalhães J. D. Finch C. Janssens G., 2010 Next-generation sequencing in aging research: emerging applications, problems, pitfalls and possible solutions. Ageing Res. Rev. 9: 315–323. 19900591 10.1016/j.arr.2009.10.006 PMC2878865

[bib33] McCormick M. Chen K. Ramaswamy P. Kenyon C., 2012 New genes that extend *Caenorhabditis elegans*’ lifespan in response to reproductive signals. Aging Cell 11: 192–202. 22081913 10.1111/j.1474-9726.2011.00768.x PMC4342234

[bib34] McGee M. D. Weber D. Day N. Vitelli C. Crippen D., 2011 Loss of intestinal nuclei and intestinal integrity in aging *C. elegans*. Aging Cell 10: 699–710.21501374

[bib35] McKinney W., 2011 Pandas: a foundational Python library for data analysis and statistics. Python for high performance and scientific computing. Available at http://www.dlr.de/sc/Portaldata/15/Resources/dokumente/pyhpc2011/submissions/pyhpc2011_submission_9.pdf. Accessed: August 10, 2017.

[bib36] Morsci N. S. Haas L. A. Barr M. M., 2011 Sperm status regulates sexual attraction in *Caenorhabditis elegans*. Genetics 189: 1341–1346. 21968192 10.1534/genetics.111.133603 PMC3241412

[bib37] Mortazavi A. Williams B. A. McCue K. Schaeffer L. Wold B., 2008 Mapping and quantifying mammalian transcriptomes by RNA-Seq. Nat. Methods 5: 621–628. 18516045 10.1038/nmeth.1226 PMC13303166

[bib38] Murphy C. T. McCarroll S. A. Bargmann C. I. Fraser A. Kamath R. S., 2003 Genes that act downstream of DAF-16 to influence the lifespan of *Caenorhabditis elegans*. Nature 424: 277–283. 12845331 10.1038/nature01789

[bib39] Murray J. I. Boyle T. J. Preston E. Vafeados D. Mericle B., 2012 Multidimensional regulation of gene expression in the *C. elegans* embryo. Genome Res. 22: 1282–1294. 22508763 10.1101/gr.131920.111 PMC3396369

[bib40] Oliphant T. E., 2007 SciPy: open source scientific tools for Python. Comput. Sci. Eng. 9: 10–20.

[bib41] Pérez F. Granger B., 2007 IPython: a system for interactive scientific computing Python: an open and general- purpose environment. Comput. Sci. Eng. 9: 21–29.

[bib42] Pimentel H. Bray N. L. Puente S. Melsted P. Pachter L., 2017 Differential analysis of RNA-seq incorporating quantification uncertainty. Nat. Methods 14: 687–690. 28581496 10.1038/nmeth.4324

[bib43] Rangaraju S. Solis G. M. Thompson R. C. Gomez-Amaro R. L. Kurian L., 2015 Suppression of transcriptional drift extends *C. elegans* lifespan by postponing the onset of mortality. eLife 4: 1–39. 10.7554/eLife.08833 PMC4720515 26623667

[bib44] Reece-Hoyes J. S. Deplancke B. Shingles J. Grove C. A. Hope I. A., 2005 A compendium of *Caenorhabditis elegans* regulatory transcription factors: a resource for mapping transcription regulatory networks. Genome Biol. 6: R110. 16420670 10.1186/gb-2005-6-13-r110 PMC1414109

[bib45] Rezával C. Nojima T. Neville M. Lin A. Goodwin S., 2014 Sexually dimorphic octopaminergic neurons modulate female postmating behaviors in *Drosophila*. Curr. Biol. 24: 725–730. 24631243 10.1016/j.cub.2013.12.051 PMC7613681

[bib46] Schedl T. Kimble J., 1988 fog-2, a germ-line-specific sex determination gene required for hermaphrodite spermatogenesis in *Caenorhabditis elegans*. Genetics 119: 43–61. 3396865 10.1093/genetics/119.1.43 PMC1203344

[bib47] Schindler A. J. Baugh L. R. Sherwood D. R., 2014 Identification of late larval stage developmental checkpoints in *Caenorhabditis elegans* regulated by insulin/IGF and steroid hormone signaling pPathways. PLoS Genet. 10: 13–16. 10.1371/journal.pgen.1004426 PMC4063711 24945623

[bib48] Seidel H. S. Kimble J., 2011 The oogenic germline starvation response in *C. elegans*. PLoS One 6: e28074. 22164230 10.1371/journal.pone.0028074 PMC3229504

[bib49] Stroustrup N. Ulmschneider B. E. Nash Z. M. López-Moyado I. F. Apfeld J., 2013 The *Caenorhabditis elegans* lifespan machine. Nat. Methods 10: 665–670. 23666410 10.1038/nmeth.2475 PMC3865717

[bib50] Sulston J. E. Brenner S., 1974 The DNA of *Caenorhabditis elegans*. Genetics 77: 95–104. 4858229 10.1093/genetics/77.1.95 PMC1213121

[bib51] Sulston J. E. Horvitz H. R., 1977 Post-embryonic cell lineages of the nematode, *Caenorhabditis elegans*. Dev. Biol. 56: 110–156. 838129 10.1016/0012-1606(77)90158-0

[bib52] Sulston J. E. Schierenberg E. White J. G. Thomson J. N., 1983 The embryonic cell lineage of the nematode *Caenorhabditis elegans*. Dev. Biol. 100: 64–119. 6684600 10.1016/0012-1606(83)90201-4

[bib53] Van Der Walt S. Colbert S. C. Varoquaux G., 2011 The NumPy array: a structure for efficient numerical computation. Comput. Sci. Eng. 13: 22–30.

[bib54] Wang C. Gong B. Bushel P. R. Thierry-Mieg J. Thierry-Mieg D., 2014 The concordance between RNA-seq and microarray data depends on chemical treatment and transcript abundance. Nat. Biotechnol. 32: 926–932. 25150839 10.1038/nbt.3001 PMC4243706

[bib55] Waskom M. St-Jean S. Evans C. Warmenhoven J. Meyer K., 2016 Seaborn: v0.7.0 (January 2016). Available at: https://zenodo.org/record/45133#.WYSHRYjytaQ. Accessed: August 10, 2017.

[bib56] Xue L. Noll M., 2000 *Drosophila* female sexual behavior induced by sterile males showing copulation complementation. Proc. Natl. Acad. Sci. USA 97: 3272–3275. 10725377 10.1073/pnas.060018897 PMC16228

